# Functional and Aesthetic Tragal Reconstruction in the Age of Mobile Electronic Devices

**DOI:** 10.1155/2016/2591705

**Published:** 2016-12-22

**Authors:** Colleen F. Perez, Curtis W. Gaball

**Affiliations:** Naval Medical Center San Diego, San Diego, CA, USA

## Abstract

We present a method to create a tragus using the patient's conchal cartilage. It is a simplified, single-stage technique with well-hidden incisions, yet it maintains the rigidity of a natural tragus. This patient did not have a history of radiation to the area, which may compromise healing with this technique. The cosmetic importance of the tragus has been described, but its functionality in accommodating modern technology has not been previously discussed. The main treatment goal for this patient was to gain the ability to wear earphones (clinical question/level of evidence: therapeutic, V).

## 1. Introduction

Various techniques for tragal reconstruction have been described primarily in the setting of microtia repair. Methods for reconstruction after tumor excision have been reported using the concha, lobule, costal cartilage, and various flaps [1–4]. The goals of tragus reconstruction in the past were only to create a pretragal depression and good tragus projection, hide the external meatus, and leave an inconspicuous scar [5].

## 2. Case Report

**Figure 1:**
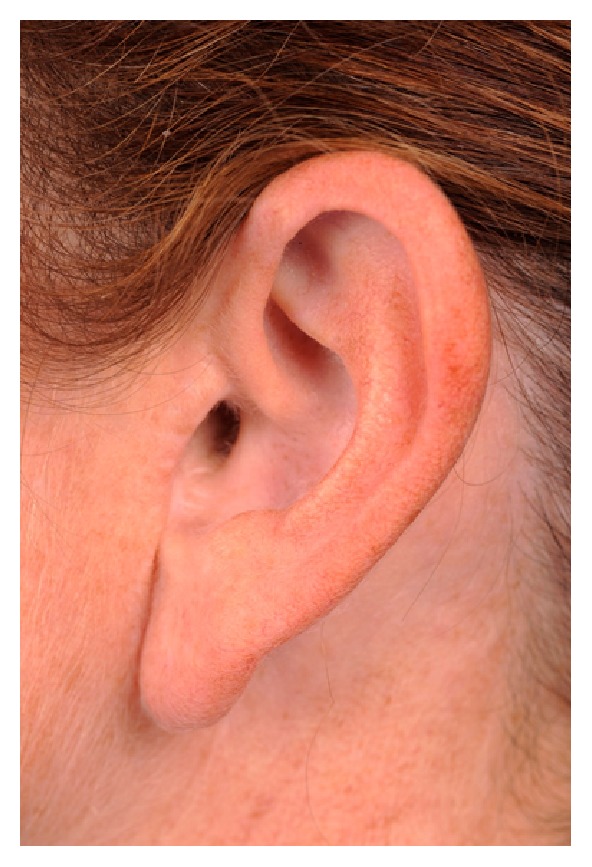
Preoperative photograph of a left tragus defect several years after excision for basal cell carcinoma.

A 43-year-old female presented with a surgically absent left tragus several years after Mohs excision for basal cell carcinoma. The original defect was closed primarily. A small remnant of the tragus remained after the original excision. She was having difficulty wearing earphones and earplugs and desired an improved appearance. She brought examples of the types of earphones she hoped to wear for surgical planning. Her preoperative photo is shown in Figure 1.

A single-stage reconstructive procedure was performed. A preauricular incision was made in a standard rhytidectomy-type configuration. The residual tragal cartilage was exposed and dissection was made on both surfaces, exposing approximately 7 mm on either side. This involved elevating off the canal skin posteriorly and the pretragal soft tissue anteriorly.

**Figure 2:**
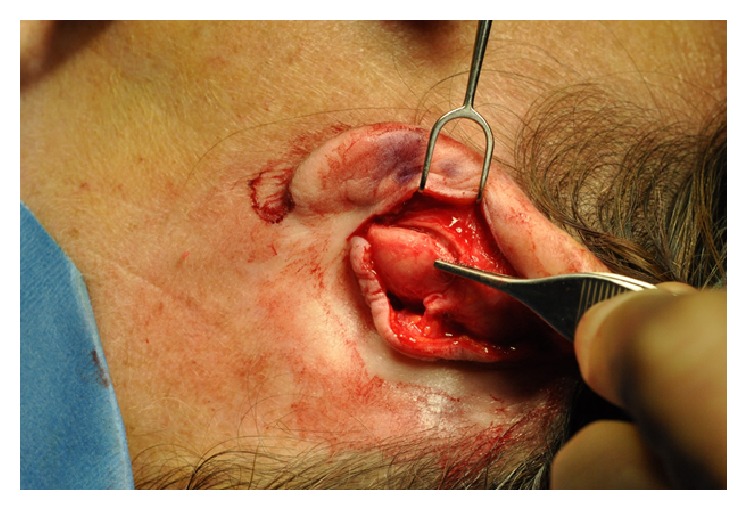
Intraoperative photograph showing conchal cartilage harvest through a postauricular incision.

**Figure 3:**
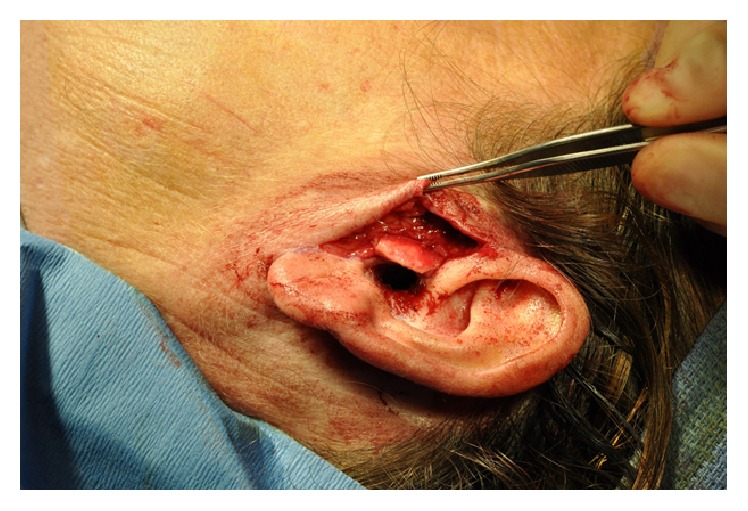
Intraoperative photograph showing the cartilage graft placement into the pretragal pocket.

Care was taken not to dissect past the tragal pointer in order to avoid facial nerve injury. The anticipated size of the new tragal cartilage and its shape were designed using the opposite ear as a guide. An area with a cartilaginous contour similar to that required was identified in the ipsilateral conchal bowl, and this segment of cartilage was harvested through a postauricular incision (Figure 2). Key landmarks of the concha were left intact, including the rim and helical root, in order to avoid any stigmata of surgery. The graft was trimmed and placed in the pretragal pocket adjacent to the residual tragal cartilage (Figure 3).

It was sutured to this residual cartilage with horizontal mattress sutures of 4-0 polydioxanone (PDS). The pretragal skin was then undermined in the subcutaneous fat plane for a distance of approximately 3 cm. The skin flap was advanced posteriorly, and the advanced skin was sutured into this new position with 4-0 PDS deep sutures to the platysma auricular fascia (PAF) to take any tension off the neotragus upon closure. The new tragal and pretragal skin was defatted as is commonly performed in facelift surgery and wrapped around the lateral surface where it met with the canal skin. Cotton soaked in mineral oil can be used as packing to coapt the overlying skin to the cartilage if skin tenting occurs. This resulted in a natural appearing pretragal depression. The closure to the canal skin was approximated with 4-0 plain gut suture. Residual skin from the advancement in the preauricular area anterior to the lobule and helix was excised away, and the remaining incision was closed with 5-0 monofilament polypropylene (Prolene) suture. The site was dressed with antibiotic ointment-soaked cotton placed in the ear canal, and a standard dental roll bolster dressing was applied to the concha and secured with a transauricular Prolene suture.

**Figure 4:**
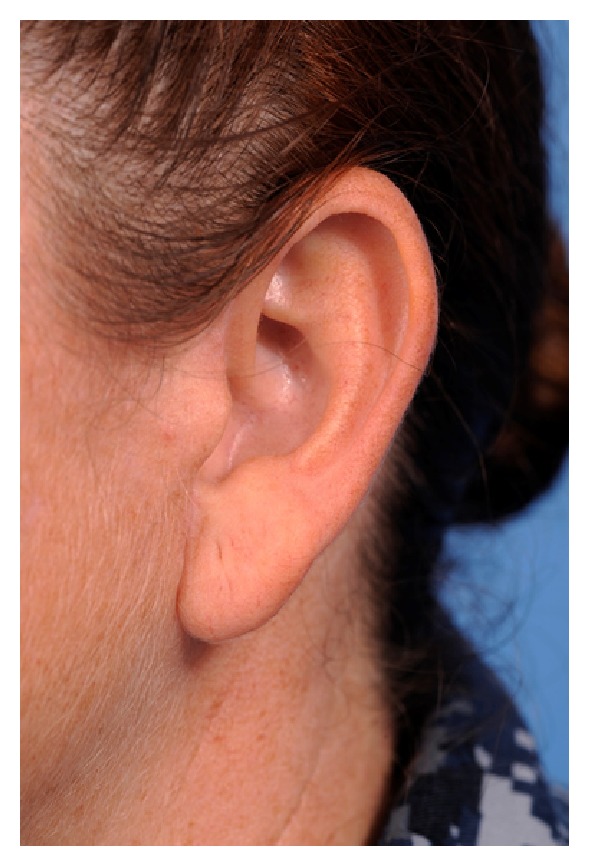
Postoperative photograph of the patient after 9 months.

The patient returned for follow-up and postoperative photos at 1 week and again at 1, 9 (Figure 4), and 13 months. At the last follow-up she reported being very pleased with her appearance and with her ability to wear earphones.

## 3. Discussion

Few reports exist describing isolated tragus reconstruction after excision of tumor in a nonirradiated field. Martínez et al. [6] reported a single-stage method using a transposition flap rotated 180 degrees based on the earlobe to reconstruct the tragus, creating projection and hiding the auricular canal. Adler et al. [5] also described a single-stage transposition flap from the preauricular area, taking care to restore the pretragus depression and create good projection. Coombs and Lin [7] used a two-stage procedure with a chondrocutaneous transposition flap from the posterior aspect of the conchal bowl. The flap is based inferiorly and transposed anteriorly as an interpolation flap. It is then divided 10 weeks later with inset of the pedicle. Scars are hidden in the postauricular sulcus, and tragal projection is maintained. No other reports of a similar single-stage conchal cartilage reconstruction for isolated tragus defect have been described. Regardless of the surgical techniques previously reported, complications related to contraction of the skin flap or distortion of the repair with time are not described.

We describe a method of a lasting, sturdy reconstruction of the tragus using ipsilateral conchal cartilage, harvested through a postauricular incision. It is a simplified, single-stage technique with well-hidden incisions and a functional result. The various techniques that have previously been described may also create excellent cosmetic results; however, more complicated or multistage methods are not always necessary. The stronger and thicker rib cartilage is most often used for microtia repair; however, for isolated tragus reconstruction, conchal cartilage has the advantages of similar character, less harvest morbidity, and faster harvest. The preauricular skin quality and laxity can be assessed by palpation, especially in those patients where there is concern for preauricular scarring or radiation damage, which may preclude them from this technique. Older individuals, who are the most common patients for Mohs, often have a large degree of excess thin skin in the preauricular area that is adequate for graft coverage. This feature makes folding the pretragal skin over the cartilage an excellent option for repair as well as for creating a tension-free closure. There should be no tension on the repair in order to prevent the reconstructed tragus from becoming distorted. The ease of using the ipsilateral ear for graft material without a distant harvest site is another advantage of this technique.

Care was taken to ensure preservation of the pretragal depression to avoid blunting of the tragus, a well-known complication after rhytidectomy [8]. Tanzer [2] described a method of creating a tragal prominence by placing a disc of cartilage through an incision behind the lobule into a tragal pocket and affixing it with compression suture. Addressing the aesthetic importance of the tragus has also been described such as the method by Chin et al. [9] in microtia repair that used a cartilage cube under the tragus for better projection and to enhance the conchal depth.

The tragus has gained a new functional importance in the modern age, since earphones are generally engineered to be reliant on it in order to maintain position. The inability to wear earphones and earplugs was the patient's biggest complaint regarding the defect after Mohs excision. With the common and growing reliance on mobile devices and technologies, which are used with earphones, this type of defect could be considered a disability. This method is a simple way to reconstruct the tragus and restore a patient's ability to wear earphones, earplugs, or hearing aids with minimal morbidity and well-hidden scars.
